# Clonal diversity and conservation genetics of the medicinal plant *Carapichea ipecacuanha* (Rubiaceae)

**DOI:** 10.1590/S1415-47572009005000096

**Published:** 2010-03-01

**Authors:** Luiz Orlando de Oliveira, Bruna Afonso Venturini, Ana Aparecida Bandini Rossi, Saulo Santos Hastenreiter

**Affiliations:** Instituto de Biotecnologia Aplicada à Agropecuaria, Universidade Federal de Viçosa, Viçosa, MGBrazil

**Keywords:** clonal propagation, genetic diversity, ISSR, poaia, *Psychotria ipecacuanha*

## Abstract

The roots of the understorey shrub *Carapichea ipecacuanha* (ipecac) have medicinal properties, and the uprooting of wild plants has supplied most of the world demand for this species. Although under severe population decline, *C. ipecacuanha* lacks legal protection. In the wild, the aerial stems of ipecac clump together to form clusters with well-defined borders. Cluster size may range from several to hundreds of aerial stems. To investigate the extent of clonality among aerial stems in ipecac clusters, we sampled 50 wild clusters (a total of 291 aerial stems) and screened them with 89 inter-simple sequence repeat (ISSR) markers. The 291 aerial stems were grouped into 42 putative clones. The clonal groups generally consisted of aerial stems from the same cluster, and there was little or no genetic differentiation among aerial stems at the cluster level. These findings suggest that strategies designed to conserve ipecac *in situ* should not rely upon census data, which are based on the number of aerial stems per cluster and the number of clusters per population, because such data greatly underestimate the species effective population size and genetic diversity. Our results also indicate that this species needs protection at a federal level.

## Introduction

Clonality is an important mechanism used by many plant species to recruit new individuals (Eriksson, 1993). In this phenomenon, the number of genets (a set consisting of all genetically identical members of a clone) may not be comparable with the number of ramets (a potentially self-sufficient unit that is a member of a genet) because many ramets may be derived from a single genet and are therefore genetically identical (Cook, 1983). Genetic diversity depends on the effective population size, defined as the number of breeding individuals in an ideal population that show the same amount of dispersion of allele frequencies under random genetic drift or the same amount of inbreeding as the population under consideration (Wright, 1931). Effective sizes in real biological systems are therefore much smaller than census sizes (Whitlock and McCauley, 1999) and cannot be predicted by merely counting the number of individuals present in a certain area because many of them may represent ramets derived from a single genet (Balloux *et al.*, 2003; Yonezawa *et al.*, 2004).

*Carapichea ipecacuanha* (Brot.) L. Andersson [= *Psychotria ipecacuanha* (Brot.) Stokes; *Cephaelis ipecacuanha* (Brot.) A. Rich.] (Rubiaceae), also known as ipecac, is a medicinal species that was widely used in traditional medicine by native Brazilians long before the arrival of European settlers (Veloso, 1947). This species is a long-lived, perennial shrub that grows in shaded understory (Veloso, 1947; Oliveira and Martins, 1998). Commercial harvesting of ipecac has occurred in Brazil since the 18^th^ century when the roots became a valuable trading good. At that time, harvesting was uncontrolled and there was no replanting after the uprooting of native populations (Oliveira and Martins, 1998). Deforestation reduced the availability of suitable habitats and accelerated even further the demographic decline of wild populations of ipecac. Today, the gathering of wild plants still provides most of the world demand for this species, although a small fraction of this demand is supplied by commercial cultivation in India, where domestication has been achieved with clones introduced from Brazil (Oliveira and Martins, 1998).

There are several indications that clonality may play a significant role in the reproduction of ipecac. Firstly, ipecacs are rarely found as isolated plants under natural growth conditions; instead, aerial stems clump together to form circular or elliptical clusters with well delimited borders. Secondly, excavations have shown that nearby aerial stems within a cluster are frequently attached by horizontal, subterranean stems and that adventitious buds located in roots and subterranean stems play a key role in promoting horizontal spread of the cluster via vegetative propagation. Within a cluster, close neighbors frequently have overlapping or interconnected underground parts and show indications of natural fragmentation and stem decay (Rossi *et al.*, 2009). Thirdly, ipecac clusters are frequently isomorphic, that is, a given cluster exhibits either short-styled or long-styled flowers (Rossi *et al.*, 2005). Clonality may have profound consequences on the dynamics and evolution of ipecac and should not be ignored in designing strategies for the conservation *in situ* of extant populations of this species. The extent of asexual propagation in ipecac should be considered carefully since estimating the number of genets based on ramet information alone (either as the number of aerial stems per cluster or the number of clusters per population) may be misleading for conservation purposes.

Dominant DNA-based markers such as AFLP, RAPD and ISSR have been used to investigate the extent of clonality in many wild species of plants. For the present study, ISSR (inter-simple sequence repeat) markers were chosen because the methodology requires no previous DNA sequence information about the target species, yields bands with greater reproducibility when compared to other DNA-based markers such as RAPD (Wolfe *et al.*, 1998), and has few requirements in terms of laboratory equipment. In this study, we investigated the extent to which aerial stems within a cluster share the same genet, and assessed the implications of clonality for the genetic conservation of ipecac *in situ*.

## Material and Methods

###  Study species

Ipecac roots have medicinal value and are used worldwide because of their expectorant, amoebicidal and emetic properties. Indeed, this species continues to be listed in the pharmacopoeias of many countries around the world (Bruneton, 1995). These activities have been confirmed pharmacologically and the isoquinoline alkaloids emetine and cephaeline have been identified as the major bioactive compounds of ipecac (Bruneton, 1995). Emetine and cephaeline accumulate in the roots throughout the year (Garcia *et al.*, 2005). As a species that is restricted to the understory, ipecac is highly sensitive to habitat changes caused by clearing, selective cutting and incidental fires that allow permanent light penetration in the canopy floor; this species also declines rapidly when exposed to forest edge environments (Oliveira and Martins, 2002). Cluster size may range from a few aerial stems to hundreds of aerial stems. Aerial stems within a cluster average 0.20 m in height and consist of about 11 nodes and up to eight totally expanded leaves (Oliveira and Martins, 2002). The contemporary populations of ipecac are confined to three clearly-defined ranges (Rossi *et al.*, 2009): (1) the Atlantic range, in the central portion of the Mata Atlântica biome along the Brazilian coast, in the states of Bahia, Espirito Santo, Rio de Janeiro, and Minas Gerais, (2) the Amazonian range, in the southwestern region of the Amazonia biome, in the Brazilian states of Rondônia and Mato Grosso, and (3) the Central-American range, the northern limit of ipecac, in Nicaragua, Costa Rica and Panama. Collections from neighboring parts of Colombia (Departments of Choco, Antioquia and Bolivar) indicate that the Central American range may extend further south. The populations of the Amazonian range in Mato Grosso are at least 2500 km from the Central-American range and 1600 km from the Atlantic range.

###  Plant materials and sampling strategy

**Figure 1:**
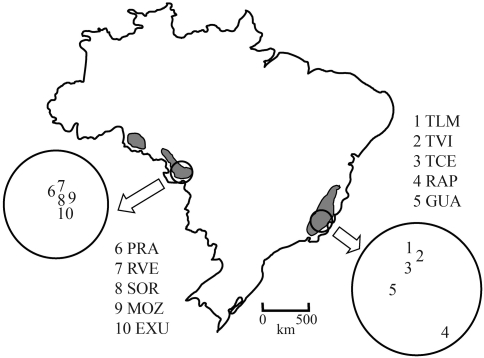
Geographic distribution and sampling locations of 10 populations of *Carapichea ipecacuanha*. Shaded regions indicate the current ranges of the species in Brazil: Amazonian range (shown at left) and Atlantic range (shown at right). The numbers refer to the approximate locations of the study populations within a given range. Refer to Table 1 for population code.

Ten populations from the two known ranges of the species in Brazil were used in this study (Figure 1). Among the five Atlantic populations, TLM, TVI and TCE were located within the Rio Doce State Park, a conservation unit that harbors extensive areas of well preserved forest; whereas RAP and GUA occurred within small forest fragments situated on privately owned land. All five Amazonian populations (EXU, MOZ, PRA, RVE and SOR) were sampled within small fragments on privately owned land in Mato Grosso. Coordinates recorded with a global positioning system (GPS) receiver were used to establish the geographic position of each population (Table 1). Five clusters were sampled in each population. On a local spatial scale, the distances between nearest clusters within a given population ranged from 30 m to 100 m. The smallest cluster was EXU-M, which occupied an area of about 1 m^2^ and contained 18 aerial stems. The largest cluster was MOZ4, which occupied an exceptionally large area (about 18 m^2^) and contained over 500 aerial stems. We avoided excavation as a mean of solving the underground connections among stems within clusters because such disturbance could harm the clusters. Moreover, since natural fragmentation and stem decay are frequent events in ipecac, two aerial stems that are currently independent of each other might have been connected in the past. As an alternative approach to increase the chances of sampling different genets within a cluster, we collected leaf samples from stems that were located on opposite sides of the cluster. A leaf sample was taken from each of six aerial stems in each cluster. Overall, young, healthy leaves from 300 stems of 50 clusters were sampled, transported to the laboratory while still fresh and stored at -80 °C until DNA extraction.

###  DNA extraction and ISSR amplification

Total genomic DNA was extracted using the method of Rossi *et al.* (2009). For ISSR amplification, a total of 100 ISSR primers (UBC primer set #9, Biotechnology Laboratory, University of British Columbia, Canada) were evaluated using two randomly chosen individuals from each of the two ranges. Twenty-four primers produced strong bands and were evaluated further for ISSR polymorphism. We tested different concentrations of several reaction components (primer: 0.15, 0.20, 0.25 or 0.33 μM; DNA template: 20, 40, 80 or 120 ng; MgCl_2_: 2.0, 2.5 or 3.0 mM; formamide: 1.5, 2.0 or 2.5%) and a range of annealing temperatures (45-56 °C). The banding pattern produced by each primer and factor combination was inspected visually for reproducibility, intensity and presence of polymorphism. Among the 24 primers, five that yielded strong, reproducible bands were selected for subsequent experiments (Table 2).

**Figure 2:**
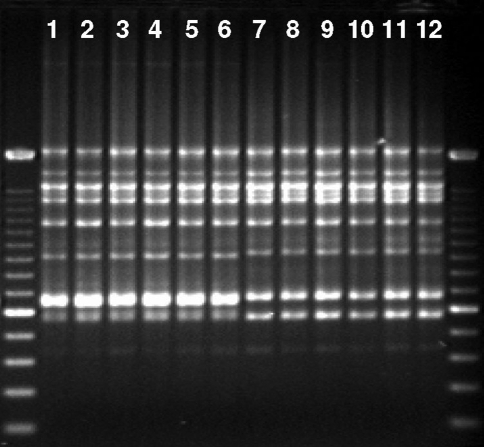
Inter-simple sequence repeat (ISSR) profiles obtained with primer UBC 834 [(AG)_8_YT]. The ISSR reaction products were electrophoresed on a 1.5% agarose gel and stained with ethidium bromide. The first and last lanes represent the 100 bp DNA ladder (Invitrogen). Lanes 1-6, aerial stems of population MOZ1; lanes 7-12, aerial stems of population MOZ2.

Final PCR amplifications were done in a total volume of 20 μL and consisted of 20 ng of template DNA, 2.0 μL of 10x PCR buffer (Phoneutria), 2.5 mM MgCl_2,_ 0.2 mM of each dNTP, 2% formamide, 0.2 μM of primer, 0.75 U of *Taq* DNA polymerase (Phoneutria) and ultrapure water. PCR amplification was programmed on a GeneAmp PCR System 9700 termocycler (Applied Biosystems) using the following conditions: a denaturation step at 94 °C for 5 min, 35 cycles of 45 s denaturation at 94 °C, 45 s annealing at 45 or 52 °C (depending upon the primer) and 90 s extension at 72 °C, followed by a 7 min extension at 72 °C. The amplification products were separated by electrophoresis on 1.5% (w/v) agarose gels with 1x TBE buffer at constant voltage (110 V) for approximately 4 h. Negative controls, in which template DNA was intentionally omitted, were used throughout the amplification. A typical gel was loaded with two sets of 12 samples and each set was flanked on both sides by a 100 bp DNA Ladder (Invitrogen). The gel was stained with ethidium bromide (0.6 ng μL^-1^) for about 30 min and then photographed under UV light using a gel documentation system (Eagle Eye II, Stratagene). To ensure consistency during scoring of band size, the images were grouped according to ISSR primer and analyzed side-by-side on a computer screen. Figure 2 shows an example of an image used to score ISSR polymorphism.

###  Data analyses

ISSR bands were treated as dominant genetic markers and scored as 1 (present) or 0 (absent). Only polymorphic bands that could be unambiguously scored across all the surveyed clusters were considered for further analysis. Six aerial stems of the Atlantic range and three aerial stems of the Amazonian range performed poorly during PCR amplifications; these samples were excluded from all subsequent analyses.

An analysis of molecular variance (AMOVA; Excoffier *et al.*, 1992) was estimated using Arlequim 3.01 (Excoffier *et al.*, 2006). AMOVA examined how genetic diversity was partitioned within and among clusters, at range and species levels. The significance of the genetic differentiation was tested with 1000 permutations, where P denotes the probability of having a more extreme variance component than the observed values by chance alone.

Cluster analyses based on unweighted pair groups with arithmetic average (UPGMA) were done using the Nei and Li (1979) genetic distance with the computer program NTSYSpc 2.2 (Rohlf, 2005) to determine the relationship among the 291 aerial stems. Neighbor-joining (NJ) analysis, as implemented in MEGA 3.1 (Kumar *et al.*, 2004), was used to construct a phenogram representing the genetic distances among the 50 clusters. This latter analysis was based on pairwise F_ST_ values taken from Arlequin 3.01 (Excoffier *et al.*, 2006). The overall fit of the NJ tree to the original distance matrix was evaluated by a cophenetic correlation coefficient implemented in NTSYS-pc 2.2 (Rohlf, 2005).

Inference of genetic structure within a given cluster of ipecac was done with a Bayesian MCMC approach, as implemented in STRUCTURE version 2.2 (Pritchard *et al.*, 2000; Falush *et al.*, 2007). In STRUCTURE, individuals (ipecac aerial stems in this case) may be members of several Bayesian groups, with the sum of membership coefficients across all groups being 1. For each population, this analysis organized the ipecac aerial stems in K groups that exhibited distinct ISSR marker frequencies (where K is chosen in advance or can be varied across different runs), with no prior information on origin. For our analysis, each class of genotypes was treated as haploid alleles, as recommended in the softwares documentation. The program was set to run the datasets from each of the ten ipecac populations as discrete inputs that were used to independently infer the genetic structure of each population. In all cases, we followed an ancestry model of admixture and a frequency model in which the allele frequencies are correlated. We set runs with a burn-in period of 20,000 and a Monte Carlo Markov chain (MCMC) of 20,000, with 10 repetitions for K = 2 to 8. STRUCTURE produced nearly identical membership coefficients at each K (data not shown) and indicated a convergence to K = 5 such that 5 (the number of cluster per population) was chosen as the best K value (Falush *et al.*, 2007).

## Results

The five ISSR primers allowed us to score 89 reproducible bands that ranged from 400 bp to about 2.1 kb, with an average of 17.8 bands per primer. For this reason, our final dataset contained a binary matrix of 291 aerial stems and 89 ISSR markers. Although the 89 bands were polymorphic at the species level, the percentage of polymorphic bands differed between ranges. Compared to the Atlantic populations, the Amazonian populations had a smaller number of bands (74 *vs.* 82) and a smaller percentage of polymorphic bands (74.5% *vs.* 93.1%) (Table 2).

The AMOVA results revealed that, at the species level, about 84% of the total molecular variance was attributable to differences among clusters, while only 16% was apportioned within clusters (Table 3). AMOVA was also used to assess population disjunction. The results revealed a similar trend at the within range level, indicating that 75%-79% of the total variance was attributable to differences among clusters within the range and 21%-25% to differences within clusters within the range.

**Figure 3:**
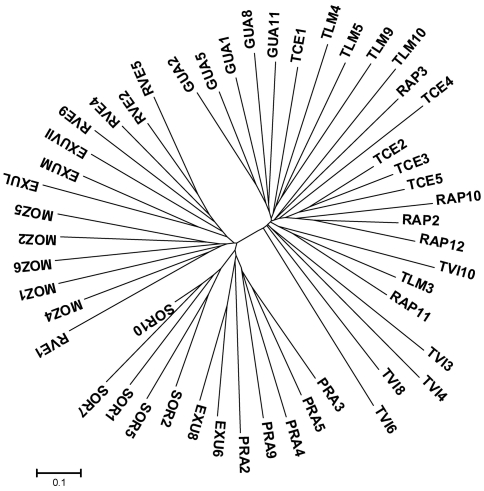
Unrooted Neighbor-Joining (NJ) tree based on pairwise FST values among clusters (taken from AMOVA) of *Carapichea ipecacuanha*. Cophenetic correlation coefficient: 0.92. Refer to Table 1 for population code.

A UPGMA dendrogram organized the 291 aerial stems into two exclusive sub-groups corresponding to the Atlantic and Amazonian ranges, and showed that most aerial stems grouped together with the remaining stems from the same ipecac cluster (data not shown). In agreement with the UPGMA dendrogram at stem level, the NJ tree (Figure 3) showed that the 50 clusters formed two exclusive sub-groups that corresponded to the Atlantic and Amazonian ranges. In most cases, a given ipecac cluster was genetically more similar to other clusters of the same population than to clusters from a distinct population.

**Figure 4:**
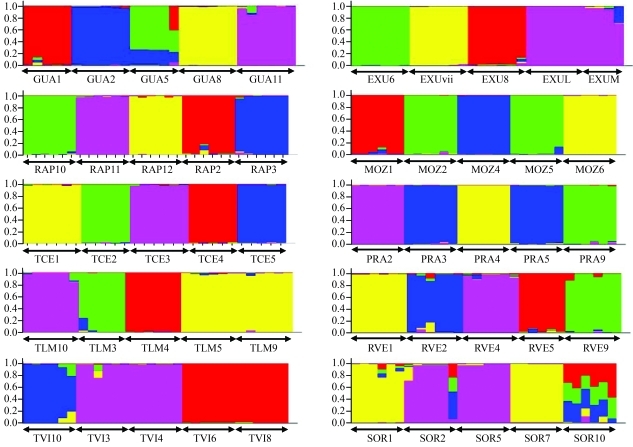
Bayesian inference of clonality in clusters of *Carapichea ipecacuanha* using the program STRUCTURE version 2.2. Each plot represents a different dataset run according to the population. In each plot, colored blocks represent the proportion of membership of an aerial steam in the inferred Bayesian group (K = 5). Each mark on the x axis represents one aerial stem. Refer to Table 1 for population code.

The results obtained with STRUCTURE provided insight into how genetic variation was partitioned among aerial stems within ipecac clusters based on the grouping of aerial stems. Here, we refrain from making inferences about the genetic relationships among aerial stems of distinct populations since the aim of this study was to assess clonality only within clusters. STRUCTURE analysis provided strong support for clonality in ipecac. The analysis revealed 42 Bayesian groups among the 50 clusters of ipecac (Figure 4). Overall, most clusters contained aerial stems with a membership coefficient in a single Bayesian group, and the aerial stems were consistently assigned to groups that contained other aerial stems from the same cluster. These results indicated that aerial stems from each group within a given population were likely to be ramets, that is, they belonged to a single genet. There were eight groups in which aerial stems from two distinct clusters were brought together and formed a single Bayesian group, *e.g.*, TLM5 and TLM9, TVI3 and TVI4, TVI6 and TVI8, EXUL and EXUM, MOZ2 and MOZ 5, PRA3 and PRA5, SOR1 and SOR7, and SOR2 and SOR5. The composition of these eight groups (Figure 4) agreed very well with the results provided by the NJ tree (Figure 3). There was only one occasion in which aerial stems of a given cluster of ipecac were split clearly into two groups: one aerial stem of cluster SOR2 and six aerial stems of cluster SOR10 formed an admixed group. The grouping a single aerial stem of SOR2 with the six aerial stems from cluster SOR10 may be considered as an illustration of a rare, recent migration event from one cluster to another.

## Discussion

The main aim of this study was to investigate the extent of genetic variation among aerial stems within clusters of ipecac. Elucidation of this issue is a crucial step towards the establishment of efficient strategies for the conservation of ipecac *in situ* since it would allow researchers and conservation agencies to include clonality (if of any significance) in the decision making process. Our initial expectation was that clonality would to some extent be present within clusters. This anticipation was based on several characteristics of ipecac, such as the clumpy growth habit, the existence of extensive underground connections among subterranean stems, the occurrence of isomorphic clusters in this distylous species, and the low rates of seed production and seed germination (Oliveira and Martins, 2002; Rossi *et al.*, 2005, 2009).

ISSR markers proved to be efficient in identifying genetically related aerial stems within a cluster of ipecac and validated our initial anticipation about the presence of clonality in this plant species. For most clusters, our molecular data showed that aerial stems were part of a single genet, as they displayed identical or near identical membership coefficients. We observed that many aerial stems had minor membership coefficients alongside a major value (shown for each mark on the x axis of Figure 4). There are at least two explanations for these findings. It is plausible that some of these small membership coefficients were actually artefacts that appeared at some point in the ISSR amplification step and during the scoring of polymorphic ISSR bands. In this case, these minor membership coefficients may be disregarded and the aerial stems from that clusters should be considered as ramets. However, it is also conceivable that these minor membership coefficients were real and arose, for example, from ISSR polymorphism that appeared because of the accumulation of somatic mutations in a perennial species (Klekowski Jr, 1997). Assuming that the minor membership coefficients were real, the aerial stems would then not belong to a single genet but to ramets derived from genets that harbored minor genetic differences. Regardless of which of the two explanations is accepted, our results clearly show that genetic differentiation among aerial stems of Brazilian ipecac was either inexistent or was extremely low at the cluster level. We have refrained from making any inferences about the genetic variation among aerial stems within clusters of Central American ipecacs because we did not have the necessary data for such a comparison. However, based on the results described here for Brazilian ipecacs, we predict that the levels of clonality within clusters of Central American ipecacs are likely to be very high.

The population sizes of Brazilian ipecacs are consistently small and the species is rare both locally and regionally. Most of the ipecac populations that we located during collecting expeditions in the Amazonian and Atlantic ranges consisted of only a handful of clusters and few populations had more than six clusters. RAP with only 17 ipecac clusters, for example, was the largest population we found in the Atlantic range (Rossi *et al.*, 2009). We are aware that our study was not exhaustive and that RAP together with populations from other well conserved forests, such as TVI, TLM and TCE, which are located in the Rio Doce State Park, may contain a larger number of clusters. Again, the scarcity of data precluded us from making inferences about the size of ipecac populations in the Central American range.

That clusters of Brazilian ipecac lack genetic diversity is important for *in situ* conservation programs. Our results indicate that the level of genetic diversity is not directly related to cluster size. From a conservational point of view, the fact that large size clusters with more than 500 aerial stems, such as MOZ4, may actually be genetically uniform or near so is worrisome. Our results also indicate that distinct clusters from a given population may actually belong to the same genet. This conclusion was based on the finding that, in eight instances, aerial stems from two different clusters were brought together into a common inter-cluster Bayesian group. Consequently, the use of census data alone, which are based on the number of aerial stems per clusters and number of clusters per population, greatly underestimates the effective population size and genetic diversity in ipecac.

Historical records (Veloso, 1947; Oliveira and Martins, 1998) indicate that ipecac was common in the wild, therefore suggesting that the current rareness may be of a recent origin. This rareness may be related to the population decline caused by commercial harvesting of wild plants in the past and may be maintained by the high level of habitat destruction and degradation (Oliveira and Martins, 2002). An alternative, but non-exclusive, explanation for the present rareness of ipecac may be that pollination biology interacts negatively with clonality. A previous study indicated that ipecac is a distylous species with isomorphic clusters and a low degree of intramorph compatibility (Rossi *et al.*, 2005). If clonality is as extensive as the present results suggest, then pollination produced by same-cluster stems is genetically equivalent to self-pollination. These features suggest that fruit production (and therefore, sexual reproduction) may rely heavily on legitimate pollination such that in a population with a 1:1 equilibrium ratio of floral morphs about half of the clusters may be unavailable as donors for cross-pollination between morphs. If, for any reason, the population is not at equilibrium, then the number of donor clusters may be even smaller. Based on these scenarios, conservationists should consider the possibility that the effective population size in ipecac is reduced even further. Additionally, the sexual reproduction of ipecac may depend upon external, ecological factors. For example, the present levels of destruction and degradation of ipecac habitats may negatively affect the availability of pollinators for the legitimate pollination of ipecac, although this remains to be investigated.

The levels of genetic diversity within Central American ipecacs and of genetic differentiation between Central American ipecacs and Brazilian ipecacs are unknown. Given the continental distances involved, high levels of genetic differentiation among the three ranges are plausible. Indeed, genetic differentiation between Amazonian and Atlantic ipecacs was high (Rossi *et al.*, 2009). These two Brazilian ranges constitute distinct centers of genetic diversity for the species. If our expectation that the Central American ipecacs are genetically distinct from Brazilian ipecacs is confirmed, then an additional, third center of diversity should exist.

Currently, most of the genetic diversity of Brazilian ipecacs is not secure within protected areas. We are unaware of the presence of ipecac in any conservation unit within the Amazonian and Atlantic ranges, the Rio Doce State Park being the only exception. An aggravating factor is that the current rates of exposure to genetic erosion and population loss are very high (Oliveira and Martins, 2002). A lack of genetic variation, small population sizes and a high risk of genetic erosion mean that ipecac is very susceptible to extinction and conservation measures are urgently needed. The species is listed as endangered by the State of Minas Gerais, the only Brazilian state within the geographic range of ipecac for which a plant conservation list has been published (Mendonça and Lins, 2000). The conservation of ipecac *insitu* will be more effective in securing genetic diversity when populations from both ranges are protected. Ipecac needs protection status at a federal level.

## Figures and Tables

**Table 1:** Ranges and populations of *Carapichea ipecacuanha* with abbreviations, sample size for data analyses (N) and geographic location.

Ranges and populations	N	Latitude (S)	Longitude (W)
Atlantic range	144		
Guaraciaba (GUA)	28	20°34'14''	43°00'27''
Raposo (RAP)	30	21°06'40''	42°05'43''
Trilha da Central (TCE)	27	19°33'44''	42°37'57''
Trilha da Lagoa do Meio (TLM)	29	19°38'34''	42°30'56''
Trilha do Vinhático (TVI)	30	19°45'44''	42°37'57''

Amazonian range	147		
Exu (EXU)	28	15°40'41''	57°32'03''
Mozar (MOZ)	30	15°04'57''	57°58'00''
Prata (PRA)	30	15°30'26''	58°01'50''
Rio Vermelho (RVE)	29	15°17'38''	57°51'43''
Soroteca (SOR)	30	15°31'34''	58°00'42''

**Table 2:** Primers used for ISSR amplification with the melting temperature (Tm), number of bands (NB) and percentage of polymorphic bands (PPB).

Primer	Sequence (5'to 3')	Tm (°C)	NB (PPB)		
			Species	Atlantic range	Amazonian range
UBC 834	(AG)_8_YT	45	12 (100)	12 (100)	8 (50)
UBC 866	(CTC)_6_	52	18 (100)	15 (93.3)	15 (73.3)
UBC 880	(GGAGA)_3_	45	18 (100)	18 (83.3)	13 (61.5)
UBC 848	(CA)_8_RG	52	19 (100)	16 (93.8)	16 (87.5)
UBC 873	(GACA)_4_	45	22 (100)	21 (95.2)	22 (100)
Total			89 (100)	82 (93.1)	74 (74.5)

*Y = C or T; R = A or G.

**Table 3:** Analysis of molecular variance (AMOVA) for different hierarchical analyses of *Carapichea ipecacuanha* populations.

Source of variation	d.f.	Sum of squares	Variance components	% total variance	p-value*
Among clusters	49	4049.725	13.4451	84.2	< 0.001
Within clusters	242	624.500	2.51815	15.8	< 0.001
Total	291	4674.225	15.96320		
Analysis within the Atlantic range					
Among clusters	24	1368.933	9.01670	75.4	< 0.001
Within clusters	123	367.333	2.93867	24.6	< 0.001
Total	147	1736.267	11.95537		
Analysis within the Amazonian range					
Among clusters	24	1140.218	7.67341	78.6	< 0.001
Within clusters	120	257.167	2.09079	21.4	< 0.001
Total	144	1397.385	9.76420		

*p-values are the probabilities of having a more extreme variance component than the observed values by chance alone. Probabilities were calculated based on 1000 random permutations.

## References

[Ballouxetal2003] Balloux F., Lehmann L., de Meeûs T. (2003). The population genetics of clonal and partially clonal diploids. Genetics.

[Bruneton1995] Bruneton J. (1995). Pharmacognosy, Phytochemistry, Medicinal Plants.

[Cook1983] Cook R.E. (1983). Clonal plant populations. Am Sci.

[Eriksson1993] Eriksson O. (1993). Dynamics of genets in clonal plants. Trends Ecol Evol.

[Excoffieretal1992] Excoffier L., Smouse P.E., Qualtro J.M. (1992). Analysis of molecular variance inferred from metric distances among DNA haplotypes: Application to human mitochondria DNA restriction sites. Genetics.

[Excoffieretal2006] Excoffier L., Laval G., Schneider S. (2006). Arlequin v. 3.01.

[Falushetal2007] Falush D., Stephens M., Pritchard J.K. (2007). Inference of population structure using multilocus genotype data: Dominant markers and null alleles. Mol Ecol Notes.

[Garciaetal2005] Garcia R.M.A., Oliveira L.O., Moreira M.A., Barros W.S. (2005). Variation in emetine and cephaeline contents in roots of wild ipecac (*Psychotria ipecacuanha*). Biochem Syst Ecol.

[KlekowskiJr1997] Klekowski E.J., de Kroon H., Van Groenendael J. (1997). Somatic mutation theory of clonality. The Ecology and Evolution of Clonal Plants.

[Kumaretal2004] Kumar S., Tamura K., Nei M. (2004). MEGA3: Integrated software for molecular evolutionary genetics analysis and sequence alignment. Brief Bioinform.

[MendoncaandLins2000] Mendonça M.P., Lins L.V. (2000). Lista vermelha das espécies ameaçadas de extinção da flora de Minas Gerais.

[NeiandLi1979] Nei M., Li W.H. (1979). Mathematical model for studying genetic variation in terms of restriction endonucleases. Proc Natl Acad Sci USA.

[OliveiraandMartins1998] Oliveira L.O., Martins E.R. (1998). O Desafio das Plantas Medicinais Brasileiras: I - O Caso da Poaia (*Cephaelis ipecacuanha*).

[OliveiraandMartins2002] Oliveira L.O., Martins E.R. (2002). A quantitative assessment of genetic erosion in ipecac (*Psychotria ipecacuanha*). Gen Res Crop Evol.

[Pritchardetal2000] Pritchard J.K., Stephens M., Donnelly P. (2000). Inference of population structure using multilocus genotype data. Genetics.

[Rohlf2005] Rohlf F.J. (2005). NTSYS-pc. Numerical taxonomy and multivariate analysis system, v. 2.2.

[Rossietal2005] Rossi A.A.B., Oliveira L.O., Vieira M.F. (2005). Distyly and variation in floral traits in natural populations of *Psychotria ipecacuanha* (Brot. ) Stokes (Rubiaceae). Rev Bras Bot.

[Rossietal2009] Rossi A.A.B., Oliveira L.O., Venturini B.A., Silva R.S. (2009). Genetic diversity and geographic differentiation of disjunct Atlantic and Amazonian populations of *Psychotria ipecacuanha* (Rubiaceae). Genetica.

[Veloso1947] Veloso H.P. (1947). As condições ecológicas da *Cephaelis ipecacuanha* Rich. Mem Inst Oswaldo Cruz.

[WhitlockandMcCauley1999] Whitlock M.C., McCauley D.E. (1999). Indirect measures of gene flow and migration: F_ST_ ≠ 1/(4Nm + 1). Heredity.

[Wolfeetal1998] Wolfe A.D., Xiang Q.-.Y., Kephart S.R. (1998). Assessing hybridization in natural populations of *Penstemon* (Scrophulariaceae) using hypervariable intersimple sequence repeat (ISSR) bands. Mol Ecol.

[Wright1931] Wright S. (1931). Evolution in Mendelian populations. Genetics.

[Yonezawaetal2004] Yonezawa K., Ishii T., Nagamine T. (2004). The effective size of mixed sexually and asexually reproducing populations. Genetics.

